# Penalized likelihood for sparse contingency tables with an application to full-length cDNA libraries

**DOI:** 10.1186/1471-2105-8-476

**Published:** 2007-12-11

**Authors:** Corinne Dahinden, Giovanni Parmigiani, Mark C Emerick, Peter Bühlmann

**Affiliations:** 1 Seminar für Statistik, ETH Zürich, CH-8092 Zürich, Switzerland; 2 Competence Center for Systems Physiology and Metabolic Diseases, ETH Zürich, CH-8093 Zürich, Switzerland; 3 Departments of Oncology and Biostatistics, Johns Hopkins Schools of Medicine and Public Health, Baltimore, MD, USA; 4 Department of Physiology, Johns Hopkins School of Medicine, Baltimore, MD, USA

## Abstract

**Background:**

The joint analysis of several categorical variables is a common task in many areas of biology, and is becoming central to systems biology investigations whose goal is to identify potentially complex interaction among variables belonging to a network. Interactions of arbitrary complexity are traditionally modeled in statistics by log-linear models. It is challenging to extend these to the high dimensional and potentially sparse data arising in computational biology. An important example, which provides the motivation for this article, is the analysis of so-called full-length cDNA libraries of alternatively spliced genes, where we investigate relationships among the presence of various exons in transcript species.

**Results:**

We develop methods to perform model selection and parameter estimation in log-linear models for the analysis of sparse contingency tables, to study the interaction of two or more factors. Maximum Likelihood estimation of log-linear model coefficients might not be appropriate because of the presence of zeros in the table's cells, and new methods are required. We propose a computationally efficient ℓ_1_-penalization approach extending the Lasso algorithm to this context, and compare it to other procedures in a simulation study. We then illustrate these algorithms on contingency tables arising from full-length cDNA libraries.

**Conclusion:**

We propose regularization methods that can be used successfully to detect complex interaction patterns among categorical variables in a broad range of biological problems involving categorical variables.

## Background

One of the most striking discoveries of the genomic era is the unexpectedly small number of genes in the human genome. This amount has decreased from more than 100000 [1] to an estimated number of roughly between 20000 and 25000 [2,3], tens of thousands less than initially expected and essentially the same number as found in phenotypically much simpler organisms. A question of overriding biological significance is, how complex phenotypes of higher organisms arise from limited genomes. Part of the explanation may be that many genes undergo a process called alternative RNA splicing, which can generate many distinct proteins from a single gene.

RNA splicing is a post-transcriptional process that occurs prior to mRNA translation. After the gene has been transcribed into a pre-messenger RNA (pre-mRNA), it consists of intronic regions destined to be removed during pre-mRNA processing (RNA splicing), as well as exonic sequences that are retained within the mature mRNA. After transcription occurs the actual splicing process, where it is decided which exons are retained in the mature message and which are targets for removal. In general, exons and introns are retained and deleted in different combinations to create a diverse array of mRNAs from a common coding sequence. This process is known as alternative RNA splicing. Depending on the source, the percentage of alternatively spliced genes lies between 35% and 60% [4-10]. By screening many full-length cDNAs it is possible to record the complete cDNA from a mature RNA for the same gene again and again and a full-length cDNA library, also known as single-gene library (SGL), builds up. The library contains detailed information about how specific exon combinations go together. This information is directly related to the functional regions of the proteins as they are grouped in domains which in many cases correspond to a single exon which encodes these domains. For example a transcription factor consists of a DNA binding domain and a regulatory domain. Thus the alteration of the exon structure corresponds to an alteration in the function of this particular domain. The central premise is that a dependency in the domains points to a functional association. If domains interact functionally then their splicing should be co-regulated. And this co-regulation has direct biological significance because it shows us which variable components also interact in the expressed protein. Because the polypeptide is intricately folded and tightly packed, segments that are separated by dozens of introns in the primary transcript may encode domains that interact functionally within the protein. These domains need not be structural neighbors even in the folded protein, but may interact through electrical or van der Waals forces, effects of global conformational changes, or even associations with other proteins. Because of these intricacies, there are no inherent distance restrictions, or limits on the number of interacting sites, and separate domains may combine their functional effect in unpredictable ways.

Due to the large number of potential combinations in highly alternatively spliced genes, any library will only comprise a small portion of the total theoretically possible inventory of combinations. Statistically, this leads to sparse contingency tables in which dimensions represent exons and cells represent variants. The investigation of interactions among categorical variables where not all possible combinations are observed, means addressing a model selection problem that is challenging both inferentially and computationally.

As far as alternative splicing is concerned, there is an important reason to determine this interaction structure: searching for intrapeptide interactions in functional assays is a very difficult, open-ended problem, where statistical analysis of the splicing interaction structure in the transcriptome can simplify this task enormously by identifying the sets of interacting domains. And as more investigators become interested in this type of information, and large-scale single-gene libraries become available, there is a strong need for reliable statistical methods for analyzing the resulting datasets.

We develop different statistical methods to analyse sparse contingency tables in order to determine the underlying interaction pattern and we use graphical models to visualize these patterns. The methods are compared in a simulation study and illustrated on full-length cDNA libraries.

## Results

### Algorithm

#### General introduction to contingency tables and Log-linear Models

In this section we provide general definitions and notations.

Assume we have *q *categorical random variables or factors, *C *= {*C*_1_,..., *C*_*q*_}, where each *C*_*j *_can take on a finite number *g*_*j *_of possible values, called levels. The vector (*c*_1_,..., *c*_*q*_) represents a particular combination of levels of the joint random variable *C *= {*C*_1_,..., *C*_*q*_}. The total cardinality of *C *is $m=\prod_{j=1}^{q}g_{j}$, which corresponds to the *m *different combinations of levels (*m *= 2^*q *^when all *C*_*j *_are dichotomous, as in our splicing example).

We simplify the notation by mapping each configuration of *C *to a unique natural number *i *∈ {1,..., *m*} with a (bijective) function *f*:

*f*: (*c*_1_,..., *c*_*q*_) ↔ *i *∈ {1,..., *m*},

so we may write **c**_*i *_= (*c*_1_,..., *c*_*q*_). For *n *observations of *C*, the corresponding *q*-way contingency table has *m *cells, each listing the frequency of a particular configuration **c**_*i*_:

$$\begin{array}{cc} n_{c_{1},...,c_{q}}=n_{i}, & \sum_{i=1}^{m}n_{i}=n. \end{array}$$

A general introduction to contingency tables can be found in [11].

If the observations are independent, with *p*_*i *_the probability of sampling configuration **c**_*i*_, the distribution of the cell counts (*n*_1_,..., *n*_*q*_)^*t *^is multinomial with probability **p **= (*p*_1_,..., *p*_*q*_).

In the splicing example, we may consider the *C*_*j *_as dichotomous random variables representing *q *sites of alternative splicing, each with two levels, denoted by *c*_*j *_∈ {1, -1}, corresponding to the presence or absence of exon *j *in a transcript. The contingency table therefore has *m *= 2^*q *^cells, with each cell represented by the *q*-dimensional binary vector **c**_*i *_= (*c*_1_,..., *c*_*q*_). A log-linear model for the cell probabilities can be written the following way:

$$\log p_{i}=\beta_{\emptyset }+\sum_{l\in \{1,...,q\}}\beta_{l}c_{l}+\sum_{\begin{array}{c} j,k\\ j<k\in \{1,...,q\} \end{array}}\beta_{jk}c_{j}c_{k}+\ldots +\beta_{12...q}c_{1}c_{2}\cdots c_{q}.$$

A general log-linear model represents **p **as:

log (**p**) = **X*β***,

where ***β ***is a vector of unknown coefficients and **X **a suitable design matrix as indicated below. Let's assume that the cell probabilities are expressed in the following way:

$$\log p_{c_{1},...,c_{q}}=\delta_{\emptyset }+\delta_{c_{1}}^{C_{1}}+\ldots +\delta_{c_{q}}^{C_{q}}+\delta_{c_{1},c_{2}}^{C_{1},C_{2}}+\ldots +\delta_{c_{1},...,c_{q}}^{C_{1},...,C_{q}},$$

where *δ*_∅ _is the global mean, $\delta_{c_{1}}^{C_{1}}$ is the main effect of the first variable and only depends on the distribution of *C*_1_. Similarly $\delta_{c_{1},c_{2}}^{C_{1},C_{2}}$ is the first order interaction between the first two variables and its value only depends on the joint distribution of these two variables.

We now look for a suitable parametrization ${\tilde{X}}^{C_{i}}$ of the vector spaces spanned by the main effects $\delta^{C_{i}}$, a parametrization ${\tilde{X}}^{C_{i},C_{j}}$ for the vector spaces spanned by the first order interactions $\delta^{C_{i},C_{j}}$ and so on. To ensure identifiability, we impose constraints on these matrices and denote the resulting matrices by $X^{C_{i}}$, $X^{C_{i},C_{j}}$ and so on. The design matrix **X **finally consists of these submatrices. The constitution of the design matrix **X **for factors with two levels can directly be derived from (1). The derivation of the design matrix **X **from (3) in the case of more than two levels per factor is basically an analysis of variance (ANOVA) parametrization with poly-contrasts. Details can be found in Additional file 1 Section 1.

Sometimes we may assume a smaller model without some of the interaction terms. It is of the form as in (2) with some columns removed from the design matrix **X**. We denote matrices of the form $X^{C_{j_{1}},...,C_{j_{k}}}$ by *X*_*a*_, with $a=\{C_{j_{1}},...,C_{j_{k}}\}\subseteq C$. The corresponding subvector of ***β ***is denoted by *β*_*a*_.

#### Graphical Models

A powerful way for visualizing conditional dependencies among variables is given by a graph. A graph G=(V,ℰ) consists of a finite set V of vertices and a finite set ℰ of edges between these vertices. In our context, the vertices correspond to the different discrete random variables. We form the so-called *Conditional Independence Graph *by connecting all pairs of vertices that appear in the same generator, that is the maximal terms *a *⊆ *C *which are present in the model. To translate a vector ***β ***into a graphical model we look for *β*_*a *_≠ 0 with *β*_*b *_= 0 ∀ *a *⊂ *b *(where *b *is a strict super-set of *a *and |*a*| > 1) and we draw edges between all vertices corresponding to *a*. From this graph we can directly read off all marginal and conditional independences by the global Markov property for undirected graphs which states: if two sets of variables *a *and *b *are separated by a third set of variables *c *then *a *and *b *are conditionally independent given *c *(*a *⫫ *b*|*c*), where for three subsets *a*, *b *and *c *of V, we say *c *separates *a *and *b *if all paths from *a *to *b *intersect *c*. For details, see [12].

#### Model selection – Non-Hierarchical versus hierarchical models

In the following subsections we introduce different model selection strategies for log-linear models. We first develop an ℓ_1_-regularization model selection approach, which is then expanded to the new so-called *level*-ℓ_1_-regularization approach. In addition, different Bayesian model selection strategies, which we use for comparisons, are explained in Additional file 1 Section 2. Hierarchical models are a subclass of models such that if an interaction term *β*_*a *_is zero, then all higher order interaction terms *β*_*b *_for *b *⊇ *a *are also zero. If we consider the example above with 2 levels, this means for example that if the first order interaction coefficient *β*_*ij *_= 0 then all higher order interaction coefficients including *i *and *j *are also zero, i.e. *β*_*ijk *_= 0, ∀ *k*. While it is possible that the true underlying interaction model may not be hierarchical from a biological standpoint, a difficulty in the use of non-hierarchical models arises from the fact that they are not invariant under reparametrization. We have chosen the design matrix ***X ***with some constraints to ensure identifiability, and we used a specific, namely an orthonormal basis. In terms of ANOVA, this choice is equivalent to choosing a poly-contrast. We could have imposed different constraints or have chosen a different basis, and this would have resulted in a different design matrix ***X ***or in terms of ANOVA, a different choice of contrast. Suppose we have found an interaction vector ***β ***for one parametrization of the log-linear model and that this vector corresponds to a non-hierarchical model, meaning there is at least one lower order interaction term *β*_*a *_equal to zero, while *β*_*b *_≠ 0 for at least one *b *⊇ *a*. If we reparametrize the model, using a different design matrix, the coefficient for the model term *a *may no longer be zero. On the other hand, by reparametrizing a hierarchical model, all zero terms remain zero after reparametrization. Therefore, hierarchicity is preserved after reparametrization while non-hierarchicity depends on the parametrization. This is a distinct advantage of working within the hierarchical class. In a hierarchical model, all zero coefficients can directly be interpreted in terms of conditional independence, while this is not true for non-hierarchical models.

#### ℓ_1_-Regularized model selection

The Lasso, originally proposed by [13] for linear regression, performs regularized parameter estimation and variable selection at the same time. The Lasso estimate is defined as follows:

$${\bar{\beta }}^{\lambda }=\arg\underset{\beta }{\min}\left[\sum_{i}{\left(Y-X\beta \right)}_{i}^{2}+\lambda \sum_{j}\left|\beta_{j}\right|\right],$$

where **Y **= (*Y*_1_,..., *Y*_*n*_) is the response vector. This can also be viewed as a penalized Maximum Likelihood estimator, as $\sum_{i}{\left(Y-X\beta \right)}_{i}^{2}$ is proportional to the negative log-likelihood function for Gaussian linear regression. While the MLE for the general regression model is no longer uniquely defined and very poor in the case of more variables than observations, the Lasso estimator is still reasonable as long as *λ *> 0. For our analysis, we have a similar problem, namely that the MLE does not exist in case of zero counts in the contingency table: a detailed description of the existence of the MLE in general log-linear interaction models is given in [14]. Inspired by the Lasso, we estimate our parameter vector ***β ***by the following expression:

$${\bar{\beta }}^{\lambda }=\arg\underset{\beta }{\min}\left[-l(\beta )+\lambda \sum_{j}\left|\beta_{j}\right|\right],$$

where *l*(*β*) is the log-likelihood function $l(\beta )=\log{\mathbb{P}}_{\beta }[n]\propto \sum_{i=1}^{m}\frac{n_{n}}{n}{\left(X\beta \right)}_{n}$. This minimization has to be calculated under the additional constraint that the cell probabilities add to 1:

$$\sum_{i=1}^{m}\exp\{{\left(X\beta \right)}_{i}\}=1.$$

A problem of the optimization (4) is that the solution is no longer independent of the choice of the orthogonal subspaces *X*_*a*_. That is, if any set of orthogonal columns *X*_*a *_of **X **is reparametrized by a different orthogonal set, we get a different solution. To avoid this undesirable outcome we use a penalty that is intermediate between the ℓ_1_- and the ℓ_2_-penalty. This penalty, called group-ℓ_1_-penalty, has the following form:

$$\sum_{a\subseteq C}{\left\|\beta_{a}\right\|}_{\ell_{2}},\text{ where }{\left\|\beta_{a}\right\|}_{\ell_{2}}^{2}=\sum_{j}{\left(\beta_{a}\right)}_{j}^{2}$$

Originally, this has been proposed by [15] for the linear regression problem with factor variables. The estimator of ***β ***then becomes

$${\bar{\beta }}^{\lambda }=\arg\underset{\beta }{\min}\left[-l(\beta )+\lambda \sum_{\begin{array}{c} a\subseteq C\\ a\neq \emptyset \end{array}}{\left\|\beta_{a}\right\|}_{\ell_{2}}\right],$$

subject to the constraint in (5). By imposing a penalty function on the coefficients of the log-linear interaction terms, overfitting as it might occur by using MLE is reduced. Furthermore, the ℓ_1_-penalty encourages sparse solutions for the single components of ***β***, the group ℓ_1_-penalty encourages sparsity at the interaction level, meaning that the vector *β*_*a*_, which corresponds to the interaction term *a *is either present or absent in the model as a whole. In case of factors with only 2 levels, the group ℓ_1_-penalty and the ℓ_1_-penalty are equivalent.

For both the ℓ_1_-, and the group ℓ_1_-regularization, the parameter *λ *can be assessed by cross-validation: we divide the individual counts into a number of equal parts and in turn leave out one part for the rest to form a training contingency table with cell counts ***n***_*train*_. The solution for an array of values for *λ*, the so-called solution path, is calculated according to an algorithm described in the following *Implementation *section. The corresponding vectors of cell probabilities are denoted by *p*(${\bar{\beta }}^{\lambda }$). We then use the remainder of the cell counts ***n***_*test *_to calculate the predictive negative log-likelihood score

$$\frac{-\sum_{i=1}^{m}n_{test,i}\cdot \log\left(p_{i}({\bar{\beta }}^{\lambda })\right)}{\sum_{i=1}^{m}n_{test,i}},$$

which is proportional to the out-of-sample negative log-likelihood. This score is on the same scale when varying the number of observations and may therefore be used to compare contingency tables of the same dimension but with different numbers of cell entries. The parameter *λ *is chosen as the value which minimizes the cross-validated score in (7). We use a ten-fold cross-validation in our example.

The resulting model does not necessarily have to be hierarchical and if we consider the hierarchical model induced by this procedure, it might happen that the final model is large for example if a single high order interaction is estimated to be active. To address this, we set up an algorithm described in the next Section.

#### Level-ℓ_1_-regularized model selection

In order to prevent the procedure from choosing single high-order interactions, we alter the ℓ_1_-regularized algorithm described in the previous Section: we do not exclusively apply it to the fully saturated model but also to submodels with lower order interactions. Precisely, a model is fitted with main effects only, and the predictive negative log-likelihood score (7) is calculated for the best main effects model (level 1). The same is done for the model including all main effects and first order interactions (level 2). Proceeding accordingly, we get |*C*| log-likelihood scores corresponding to the |*C*| levels. The level with minimal score (7) is then chosen (and within this selected level, we have an ℓ_1_-regularized estimate).

With this procedure the tendency of including a single high-order interaction while most of its lower order interactions are absent is decreased, and the inclusion is only forced if the predictive negative log-likelihood score strongly speaks in favour of the inclusion. Therefore we tend to select sparser models which can be better hierarchized and interpreted in terms of conditional independence, in contrast to the ordinary ℓ_1_-model selection procedure.

#### Algorithm for ℓ_1_-regularization for factors with two levels

For the regularization approaches we calculate ${\bar{\beta }}^{\lambda }$ over a large number of values of *λ *in order to do some cross-validation using (7). For this purpose, an efficient algorithm is required. As one can easily verify by introducing Lagrange multipliers, finding the solution to (6) under the constraint (5) is equivalent to minimizing an unconstrained function *g*(***β***):

$$g(\beta )=-l(\beta )+\sum_{i=1}^{m}\exp(\mu_{i})+\lambda \sum_{\begin{array}{c} a\subseteq C\\ a\neq \emptyset \end{array}}{\left\|\beta_{a}\right\|}_{\ell_{2}},$$

with ***μ ***= **X*β ***and $l(\beta )\propto \sum_{i}\frac{n_{i}}{n}{\left(X\beta \right)}_{i}$. Here, *g *is a convex function. If each factor has two levels only, as in our application with single-gene libraries, we can set up an algorithm, which efficiently yields the estimates for a whole sequence of parameters *λ*. Let A denote the set of active interaction terms, which means for *a *∈ A it holds that *β*_*a *_≠ 0; $X_{A}$ is the corresponding sub-matrix of **X**, $\beta_{A}$ the corresponding sub-vector of ***β ***and $g_{A}$ is *g *restricted to the subspace $\beta_{A}$. We restrict ourselves to the currently active set A, where $\nabla g_{A}$ and $\nabla^{2}g_{A}$ are well-defined:

$$\begin{array}{c} \nabla g_{A}(\beta_{A},\lambda )=-X_{A}^{t}\{\frac{n}{n}-\cdot \exp(X_{A}\beta_{A})\}+\lambda {\left(0,sign(\beta_{A})\right)}^{t}\\ \nabla^{2}g_{A}(\beta_{A},\lambda )=X_{A}^{t}diag\{\exp\{X\beta )\}X_{A}. \end{array}$$

The algorithm, which is an adaption of the path following algorithm proposed by [16], is set up as follows:

(1) Start with $\bar{\beta }$ = (-log(*m*), 0,..., 0)

(2) Set: *λ*_0 _= 1, A = {∅} and *t *= 0.

(3) While (*λ*_*t *_> *λ*_*min*_)

(3.1) *λ*_*t*+1 _= *λ*_*t *_- *ε*

(3.2) A = A ∪ {*j *∉ A: |[**X**^*t*^·$\frac{n}{n}$ - exp (**X**$\bar{\beta }$)]_*j*_| > *λ*_*t*+1_}

(3.3) $\bar{\beta }$ is updated as $\bar{\beta }$_*t*+1 _= $\bar{\beta }$_*t *_- $\nabla^{2}g_{A}$($\bar{\beta }$_*t*_, *λ*_*t*+1_)^-1^·$\nabla g_{A}$($\bar{\beta }$_*t*_, *λ*_*t*+1_).

(3.4) A = A\{*j *∈ A: {$\bar{\beta }$_*t*+1,*j*_| <*δ*}

(3.5) *t *= *t *+ 1

The pairs $\left({\bar{\beta }}_{t},\lambda_{t}\right)$, obtained from the algorithm above, represent the estimates from (6) under the constraint (5) for a range of penalty parameters *λ*_*t *_e.g. (*t *= *ε*, 2*ε*...). The choice of the step length *ε *represents the tradeoff between computational complexity and accuracy. To increase accuracy, one can perform more than one Newton step (3.3) if the gradient starts deviating from zero. The coefficient *δ *is also flexible. Typically it is chosen in the order of *ε*. The lowest *λ *for which one wants the solution to be calculated is denoted by *λ*_*min*_. Technical details concerning the algorithm can be found in the Appendix.

### Testing

#### Data

We choose the true underlying interaction vector ***β ***consisting of 5 factors of 2 levels. By enumerating the factors from 1 to 5, the generators of the model are 345 + 235 + 234 + 135 + 123 + 14, which means that all third and fourth order interactions are absent, only five of ten second order interactions and all first order interactions are present. The corresponding coefficients of ***β ***are independently simulated using a normal distribution with mean zero and variance one.

Then, 250 draws from a multinomial distribution with probability vector **p **where log (**p**) = **X*β***, are taken. This corresponds to a reasonable number of cDNAs in a single-gene library. This is then repeated 10 times. With our choice of ***β***, the resulting contingency tables are sparse. With the simulated cell counts, $\bar{\beta }$ is estimated with different methods described in the previous sections and these methods are then compared as follows:

#### Criteria

As a model selection score (MSS), we consider the fraction of correctly assigned model terms:

$$\text{MSS}=1-\frac{1}{m}\sum_{i=1}^{m}\left|1_{\left\{\beta_{i}\neq 0\right\}}-1_{\left\{{\bar{\beta }}_{i}\neq 0\right\}}\right|.$$

Moreover, we consider the root mean squared error for the interaction coefficients,

$$RMSE=\sqrt{\frac{1}{m}\sum_{i=1}^{m}{\left({\bar{\beta }}_{i}-\beta_{i}\right)}^{2}}.$$

For assessing how much the estimation of ***β ***varies over multiple datasets, we calculate for every coefficient ${\bar{\beta }}_{i}$ the estimated standard deviation ${\bar{\sigma }}_{i}$. The means of these standard deviations are reported as

$$\text{SPREAD}=\frac{1}{m}\sum_{i=1}^{m}{\bar{\sigma }}_{i},$$

a measure of variability.

To compare the different procedures for estimation of probabilities **p **= exp (**X*β***), we calculate the negative log-likelihood score (NLS) similar to the score in (7):

$$\text{NLS}(\bar{\beta })=-\sum_{i=1}^{m}p_{i}\cdot \log\left(p_{i}(\bar{\beta })\right).$$

#### Results of simulation study

The results of the simulation study are summarized in Table 1, where we also include the MAP estimators of the Bayesian approaches described in Additional file 1 Section 2. We notice that the penalty-based regularization approaches proposed in this article leads to comparable or better results than the Bayesian approaches with respect to the NLS-score, RMSE and the variation (SPREAD), though the results of Bayesian approaches vary with the prior and the set of possible priors has not been extensively explored.

**Table 1:** Performance of different algorithms

	MSS	NLS	RMSE	SPREAD
Penalty-based regularization methods:				
ℓ_1_-regularization	69.7%	2.20	0.228	0.144
Level-ℓ_1_-regularization	89.7%	2.22	0.237	0.179
Relaxed ℓ_1_-regularization	82.2%	2.22	0.233	0.154
ℓ_2_-regularization	-	2.20	0.238	0.130
MCMC without model selection:				
*σ*^2 ^= 2	-	2.32	0.747	0.401
*σ*^2 ^= 1	-	2.27	0.467	0.287
*σ*^2 ^= 1/2	-	2.24	0.294	0.201
MCMC with model selection:				
*σ*^2^~Γ^-1^(2,3)	81.5%	2.23	0.294	0.231
*σ*^2 ^= 2	76.6%	2.25	0.431	0.342
*σ*^2 ^= 1	78.4%	2.24	0.331	0.265
*σ*^2 ^= 1/2	76.6%	2.23	0.281	0.225
MCMC with hierarchical model selection:				
*σ*^2^~Γ^-1^(2,3)	84.1%	2.22	0.255	0.180
*σ*^2 ^= 2	80.6%	2.29	0.415	0.284
*σ*^2 ^= 1	83.4%	2.26	0.308	0.221
*σ*^2 ^= 1/2	83.4%	2.24	0.247	0.178
*σ*^2^_1 _= 1/10	86.3%	2.20	0.236	0.097
*σ*^2 ^= 1/100	69.7%	2.28	0.420	0.033

Comparison of different methods to estimate the interaction strength vector ***β***. MSS, NLS, RMSE and SPREAD are described in the *Implementation *section. The additional methods relaxed ℓ_1_-regularization and ℓ_2_-regularization listed in the Table are explained in the Results Section.

The level-ℓ_1_-regularization and the relaxed ℓ_1_-regularization (see below) are both competitive and can be better than MCMC for model selection.

The results of the MCMC procedures are sensitive to the choice of the prior value or the prior distribution for *σ*^2^. A at prior for *α*_*a *_(*σ*^2 ^= 2) results in worse performance than that of a prior that shrinks the coefficients more towards zero (*σ*^2 ^= 1/2). This suggests that specification of this prior hyperparameter may be difficult in practice, while we can easily optimize *λ *in the regularization approach by cross-validation.

The MCMC approaches without model selection perform poorly, as should be expected from data generated by a sparse model. MCMC methods based on a non-hierarchical model selection are also clearly inferior to the hierarchical counterpart. This is not surprising, as we have simulated data from a hierarchical model. In Table 1 we have also added an additional approach, denoted by ℓ_2_, the equivalent to the ℓ_1_-regularization but using an ℓ_2_-penalty instead of an ℓ_1_-penalty on the coefficients of the log-linear model. This method is equivalent to the MAP estimator with Gaussian priors on *β*_*a*_, with the parameter of the distribution optimized by cross-validation. This Ridge-type method does not perform variable selection, but it is competitive for all other criteria that we assessed.

In addition we consider the *relaxed *ℓ_1_-regularization approach. Rather than using a single penalty parameter *λ*, the idea of this method is to control variable selection and parameter estimation by incorporating two penalty parameters. For linear regression it has been proven theoretically as well as empirically [17] that under suitable conditions the relaxed ℓ_1_-regularization is better than Lasso.

Overall, the level-ℓ_1_-regularization has good model selection performance (high MSS score) in combination with low negative log-likelihood score (NLS) and a low mean squared error for the true ***β ***(RMSE). In addition, it is feasible to optimize the tuning parameter *λ *by cross-validation as the computational cost is very low compared to the MCMC approaches. On the other hand, posterior distributions of estimates from MCMC methods provide additional information about uncertainty in the model space, compared to point estimates from ℓ_1_- or ℓ_2_-regularization.

### Implementation

#### Dataset

We estimate the splicing interaction pattern for a dataset corresponding to the *itpr1 *gene, one of three mammalian genes encoding receptors for the second messenger inositol 1,4,5-trisphosphate (InsP_3_). This gene is subject to alternative RNA splicing, with seven sites of transcript variation, 6 of these within the ORF and among these, *q *= 5 were completely assessed in the single-gene libraries. Five single-gene libraries were built, one for adult rat cerebrum as well as four for different stages of postnatal cerebellar development, namely on days 6, 12, 22 and 90, the latter being considered as adult. Each library consists of between 179 and 277 transcripts which were assessed, i.e. $\sum_{j=1}^{m}n_{j}$ ∈ [179, 277]. This gene is 89% identical at the cDNA level and 95% identical at the amino acid level with the human receptor gene. The complete dataset can be found in [18].

#### Results of application to Single-Gene Libraries

**Figure 1:**
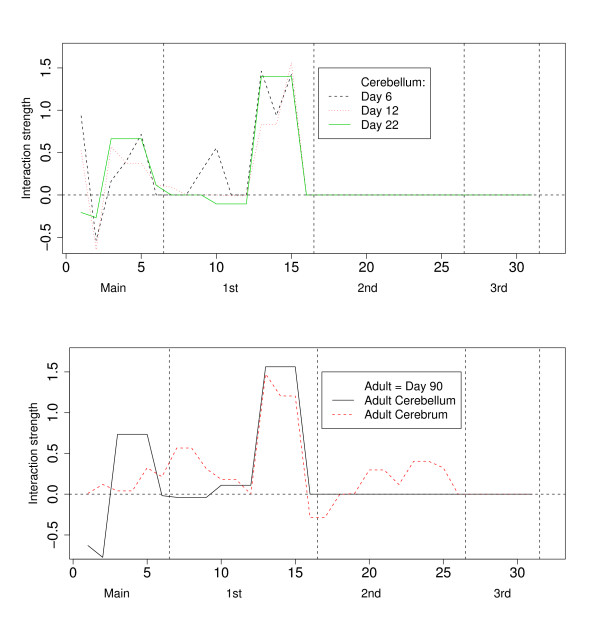
**Graphical display of interaction vector**. The upper panel shows the estimated splicing interaction vectors $\bar{\beta }$ of rat cerebellum tissues at postnatal days six, 12 and 22. The lower panel shows the splicing interaction vector $\bar{\beta }$ of rat cerebellum tissues at the age of 90 days, which is considered adult, as well as the splicing interaction vector $\bar{\beta }$ of rat cerebral tissue at the age of 90 days. Within an interaction degree, the sequence of coefficients is ordered from left to right as follows: e.g. for 2nd order interactions, 123, 124, 125,..., 345, where 1,...., 5 represent exons 12, 23B, 40, 41, and 42 in the rip3r1 gene, as described in [18].

Unless stated differently, we report the results using the level ℓ_1_-penalization method. We display the interaction vector $\bar{\beta }$ graphically by plotting the components ${\bar{\beta }}_{j}$ for the different tissue and development stages in Figure 1. Our results suggest that the exons interact mainly in pairs and there is no reliably estimated higher order interaction in the splicing interaction pattern of rat cerebellum. We further notice that the main interaction pattern is very well conserved over different developmental stages. A strong mutual interaction between exons number three, four and five can be observed in all development stages of rat cerebellum as well as in the cerebral tissue. The biggest changes in the interaction pattern during development of rat cerebellum occur from postnatal day six to day 12. This can be seen at position number 10 on the x-axis in Figure 1, and it corresponds to the first order interaction between exons two and three, and from day 12 to day 16, the first main effect changes in sign and magnitude. The first main effect decreases progressively from day 6 to adult, reversing in sign between day 12 and 22. Between day 22 and 90, the interaction pattern is strongly conserved. Comparing the splicing interaction patterns between cerebellum and cerebrum in the adult rat, we see a much more complex pattern in the cerebrum, involving several second order interactions, and therefore a clear distinction from that of the cerebellum.

**Figure 2:**
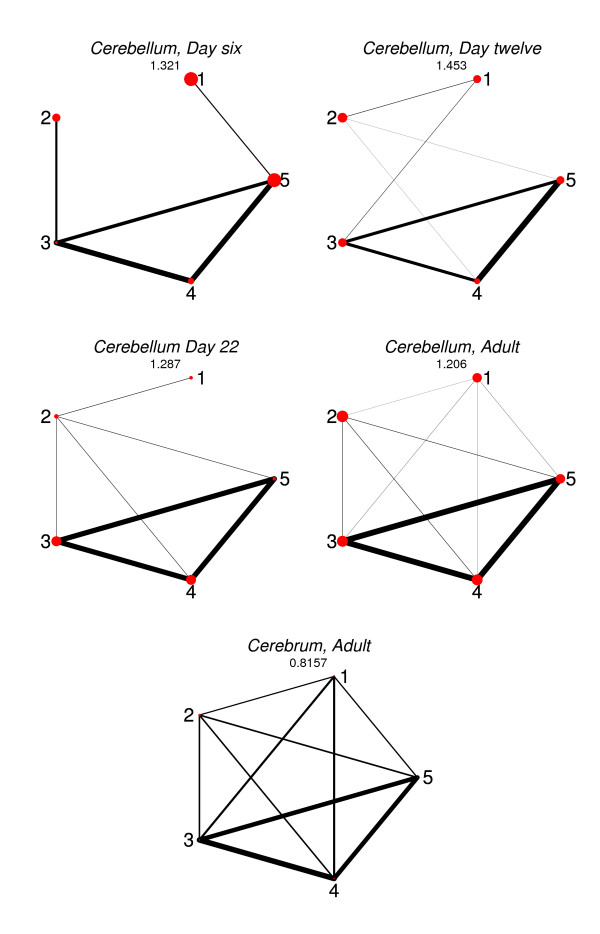
**Conditional Independence Graphs**. Conditional independence graphs for the estimated log-linear models for the *itpr1 *gene. For each graph, the predictive probability score (7) is reported as a goodness of fit measure. Note the strong mutual interaction between exons three, four and five.

The conditional independence graphs for the estimated log-linear models are drawn in Figure 2, where the thickness of the edges are proportional to the corresponding coefficient of the interaction vector $\bar{\beta }$ (the largest, if there are several giving rise to the same edge) and the radius of the vertices are chosen proportional to the corresponding main effect coefficient. Figure 2 graphically exploits the strongly conserved interactions between exons three, four and five. Except for a rather strong interaction between exon two and three on day six, all other interactions appear to be rather small. The graphical representation of the interaction pattern of adult rat cerebrum reveals a more complex interaction pattern with no conditional independences.

The approaches and results presented here can provide valuable insight into the underlying processes in alternative splicing in general, and specifically in the brain development experiments considered here. Most striking is the strong conservation over developmental stages at day 12, 22 and 90 (adult); some differences are showing between postnatal day six and day 12. Also, the conservation between the cerebellum and cerebrum is less pronounced than over developmental stages. Finally, second- or higher-order interaction terms seem to be of minor relevance, suggesting that in this gene/tissue combination, direct interaction mainly happens between pairs of exons, but not combinations of three or more exons.

We have also estimated ***β ***with the hierarchical Bayesian approach using MCMC. For the choice of *σ*^2 ^= 1 this resulted in very similar interaction patterns as for the level ℓ_1_-penalization method. For *σ*^2 ^= 2 it led to remarkably different results. In addition to this, a further dataset was analyzed where the details can be found in Additional file 1 Section 3.

## Conclusion

We have developed an efficient method for identifying interaction patterns of categorical variables. This can be used to fit a graphical model which is a valuable tool to visualize the conditional dependence structure among the random variables. In a simulation study, the results of the new level-ℓ_1_-regularization method are superior in comparison to ℓ_1_-regularization and slightly better than the MAP estimator from some of the MCMC methods we considered. With real data, the level ℓ_1_-regularization and hierarchical Bayesian approach led to similar results, subject to a specific choice of priors for the Bayesian method. An important computational advantage of the level-ℓ_1_-method in comparison to MCMC, is that cross-validation becomes feasible which in turn allows for an empirical choice of the tuning parameter. While the methodology described in this article is motivated by the study of exon splicing interactions in single-gene transcriptomes, it provides a general and flexible toolbox for regularization analysis in relatively high dimensional, sparse contingency tables. Model selection in high dimensional contingency tables has been a traditionally challenging area, and we hope that our generalization of regularization methodologies to this context will prove useful in a variety of areas of computational biology and biostatistics. Several technologies generate categorical data: these include SNP chips that provide genotype and copy number information at the DNA level, sequencing technologies, assays that study binding properties of proteins and binding of RNA to DNA, a variety of disease phenotypes, and more. In most of these contexts the interactions among the variables are critical features in systems biology investigations that aim at studying how the components of complex systems work together in in fluencing biological outcomes. For example, the log-linear models described here provide a natural approach for fitting very general classes of networks to discrete data. The level-ℓ_1_-regularization is a general tool which can be applied to a wide variety of problems involving sparse contingency tables.

An R package called *logilasso *will be available for download on the Comprehensive R Archive Network (CRAN).

## Authors' contributions

CD derived the mathematical details, implemented and tested the algorithm. GP initiated the project, suggested ideas and edited the manuscript. ME provided the datasets and the biological interpretation. PB supervised the project and suggested some of the main ideas. All authors read and approved the final manuscript.

## Appendix

We note that if ***β ***is a minimum of *g*, then $\beta_{A}$ is a minimum of $g_{A}$.

In our application with single-gene libraries, all factors have two levels only, which allows to construct an efficient algorithm. Since the gradient

$$\nabla \left[-l(\beta )+\sum_{j=1}^{m}\exp(\mu_{j})\right]=-X^{t}\cdot (\frac{n}{n}-\exp(X\beta )),$$

where exp(**X*β***) is understood as the componentwise exponential function, it follows that for a minimum $\beta_{A}$ of $g_{A}$, the following equation holds:

$$\nabla g_{A}(\beta_{A})=-X_{A}^{t}\cdot (\frac{n}{n}-\exp(X_{A}\beta ))+{\left(0,sign(\beta_{A})\right)}^{t}\cdot \lambda =0$$

Without loss of generality, we can restrict ourselves to the subspace ***β ***∈ ℝ^- ^× ℝ^*m*-1^, because the constraint (5) can only be satisfied for *β*_∅ _< 0 as is proved in the following Lemma 1. Therefore *β*_∅ _∈ A.

**Lemma 1**. *β*_∅ _< 0 *for a minimum of g*(***β***) *for all λ *∈ ℝ^+^.

*Proof*.

log(**p**) = **X*β ***< 0 which yields (1,..., 1)**X*β ***= *mβ*_∅ _< 0 this implies *β*_∅ _< 0.

This holds because (1,....., 1) is orthogonal to all columns of **X **except for the first one.   □

Additionally for ***β ***being a minimum, a necessary condition is:

$$|{\left(X^{t}\cdot (\frac{n}{n}-\exp(X\beta ))\right)}_{j}|<\lambda ,\forall_{j}\notin A.$$

Conditions (9) and (10) are sufficient for ***β ***being a minimum of (8). To find the ***β***'s that solve these equations for an array of values for *λ*, we set up a so-called path following algorithm. The idea is to start from an optimal solution $\beta^{\lambda_{0}}$ for *λ*_0_, and follow the path for decreasing *λ*, using a second-order approximation for $\beta_{A}$. In the following, we restrict ourselves to the currently active set A, omitting the index A. It then holds:

$$\begin{array}{c} \nabla g(\beta_{t+1},\lambda_{t+1})=0\approx \nabla g(\beta_{t},\lambda_{t+1})+\nabla^{2}g(\beta_{t},\lambda_{t+1})\delta \beta .\text{ This implies}\\ \delta \beta =-\nabla^{2}g{\left(\beta_{t},\lambda_{t+1}\right)}^{-1}\nabla g(\beta_{t},\lambda_{t+1}). \end{array}$$

The algorithm tries to follow the optimal path as close as possible. At each step, it aims to meet the conditions (9) and (10). In step (3.2), the active set A is identified, which forces $\bar{\beta }$ to meet the condition (10). In step (3.3), a Newton step as described in (11) is performed. Starting from a solution which meets condition (9), the new ${\bar{\beta }}^{\lambda }$ approximately meets (9) again.

## Supplementary Material

Additional file 1 The Additional file consists of 3 sections. Section 1 contains details concerning the parametrization of the log-linear model. Section 2 describes some Bayesian model selection approaches, which were used for comparison with our algorithm. In Section 3 a further dataset on which we tested our algorithm is introduced and the results are given on that dataset. Click here for file
