# Supplementary material for: Penalized likelihood for sparse contingency tables with an application to full-length cDNA libraries
Source: BMC Bioinformatics. 2007 Dec 11;8:476. doi: 10.1186/1471-2105-8-476 (PMC2233645; doi:10.1186/1471-2105-8-476)

Additional files

Additional file 1 – Parametrization of log-linear model

A possible parametrization of the vector space spanned e.g. by δ^{C_1}, $span(\delta^{C_1}) = \tilde{X}^{C_1} \in \mathbb{R}^{m\times|C_1|}$, is given as follows: the jth column takes on the value 1 if the variable c_1 of the corresponding cell takes on level j and 0 otherwise. Similarly for $\tilde{X}^{C_1,C_2} \in \mathbb{R}^{m\times(|C_1|\cdot|C_2|)}$, there is a 1 in the first column, if the corresponding cell has c_1 as well as c_2 taking on its first level. Of course the resulting matrix is highly overparametrized and to ensure identifiability, we have to impose constraints. By definition the vector space spanned by the constant vector $\delta_\emptyset$ is a subspace of the column space of $\tilde{X}^{C_1}$, analogously the column space spanned by $\tilde{X}^{C_1}$ and $\tilde{X}^{C_2}$ is a subspace of the column space spanned by $\tilde{X}^{C_1,C_2}$ and the same holds for higher order interactions: The vector space spanned by an interaction of order k is always a superspace of all vectorspaces spanned by all corresponding interactions of order $k-1$. We then finally define $X^{C_{j_1},\ldots,C_{j_k}}$ by the orthogonal complement of the vector space spanned by $\{\tilde{X}^{C_{j_2},\ldots,C_{j_k}}, \tilde{X}^{C_{j_1},C_{j_3},\ldots,C_{j_k}}, \tilde{X}^{C_{j_1},\ldots,C_{j_{k-1}}}\}$ in $\tilde{X}^{C_{j_1},\ldots,C_{j_k}}$. The dimensionality of the resulting submatrix is $\mathbb{R}^{m\times\left((|C_{j_1}|-1)\ldots(|C_{j_k}|-1)\right)}$. We can easily prove that the resulting design matrix is an orthogonal matrix. We generally normalize the column vectors to length $\sqrt{m}$, such that the first column is a column of 1's.

The log-linear interaction model (3) takes on the following form in matrix formulation:

$$\log(\mathbf{p}) = \mathbf{X}\boldsymbol{\beta}.$$

Additional file 2 – Bayesian model selection

Non-hierarchical Bayesian model selection

The Bayesian approach we choose is essentially the same as chosen by [1] and closely related to what was proposed by [2, 3, 4]. We use a Markov chain Monte Carlo algorithm based on Stochastic Search Variable Selection (SSVS): SSVS is a procedure proposed by [3] to perform variable selection in the standard linear regression model. We adapt this procedure to log-linear models. But instead of assuming a normal mixture model for the coefficients of interest as in SSVS, we follow an approach proposed by [4], and assume the coefficients to be a mixture of a point mass at zero and a normal distribution. Following the notation introduced in the main text, the complete model can be described as follows:

$$\begin{array}{c}\mathbf{n} \sim Multinom(\mathbf{p}) \text{ with } \log(\mathbf{p}) = \mathbf{X}\boldsymbol{\beta},\\ \beta_a|\gamma_a \sim (1-\gamma_a)I_0 + \gamma_a\mathcal{N}(0,\sigma_a^2 1_{d_a}) \text{ independent for all } a \subseteq C,\\ \gamma_a \sim Ber(pr_{\gamma_a}) \text{ independent for all } a \subseteq C,\\ \sigma_a^2 \sim \Gamma^{-1}(l,u) \text{ independent for all } a \subseteq C,\end{array} \quad (1)$$

where I_0 is a point mass at zero and γ_a is a Bernoulli variable with probability parameter pr_{γ_a} reflecting prior belief that the corresponding interaction term β_a is not equal to zero. The parameters σ_a^2 follow an

inverse gamma distribution with parameters l and u. In our simulation study, we also considered fixed values for σ_a^2. The choice of the prior parameter l, u and pr_{γ_a} is discussed in the *Implementation* section. In the absence of strong prior belief, it is reasonable to assume that all σ_a^2 are identically distributed. By imposing prior distributions on the log-linear parameters β_a, it would be possible to incorporate further prior knowledge in the form of existence of correlation or signs of correlation between the different criteria C. One way is to use a prior with expectation different from zero for the corresponding log-linear term ($\mathbf{E}\left[\beta_a|\gamma_a = 1\right] \neq 0$). See for example [5] for a more detailed discussion on normal priors for the log-linear parameters β_α.

We introduce variables α_a, where $\alpha_a \sim \mathcal{N}(0, \sigma_a^2 1_{d_a})$ and we set $\beta_a = \alpha_a$ if $\gamma_a = 1$ and $\beta_a = 0$ if $\gamma_a = 0$ independent of the value of α_a: $\beta_a = \alpha_a\gamma_a$ has then the desired distribution in (1). This construction is mentioned, but not implemented, in [4].

The calculation of the posterior distribution $f(\boldsymbol{\gamma}, \boldsymbol{\alpha}, \boldsymbol{\sigma}^2|\mathbf{n})$ is now required. This cannot be done directly and Monte Carlo approximations are needed, for example from Gibbs sampling. We first calculate the univariate conditional distributions of the parameters α_a or components of $\boldsymbol{\alpha_a}$ if it is a vector:

$$f(\alpha_a|\mathbf{n}, \boldsymbol{\gamma}, \boldsymbol{\alpha}_{\setminus a}, \boldsymbol{\sigma}^2) \propto f(\mathbf{n}|\boldsymbol{\gamma}, \boldsymbol{\alpha}) f(\alpha_a|\sigma_a^2) \propto \exp\left\{\mathbf{n} \cdot (X_\emptyset \alpha_\emptyset + X_a \alpha_a \gamma_a)\right\} f(\alpha_a|\sigma_a^2).$$

Although this univariate conditional density is not of any recognized form, we can prove that it is log-concave (see Lemma 1 at the end of this section for details) and therefore sampling from it can be efficiently done using adaptive rejection sampling, as proposed by [6]. Sampling σ_a^2 is straightforward, as

$$f(\sigma_a^2|\mathbf{n}, \boldsymbol{\gamma}, \boldsymbol{\alpha}, \boldsymbol{\sigma}^2_{\setminus a}) = f(\sigma_a^2|\alpha_a) \propto f(\alpha_a|\sigma_a^2) f(\sigma_a^2), \tag{2}$$

and we can easily show that $\sigma_a^2|\alpha_a \sim \Gamma^{-1}(\alpha_a^2/2 + l, u + 1/2)$. Therefore we can sample σ_a^2 from an inverse gamma distribution. In the case where σ_a^2 is assumed to be fixed, this sampling step can be omitted. To sample from $f(\gamma_a|\mathbf{n}, \boldsymbol{\gamma}_{\setminus a}, \boldsymbol{\alpha}, \boldsymbol{\sigma}^2)$, we compute the conditional Bayes factor BF in favour of $\gamma_a = 1$ versus $\gamma_a = 0$. The conditional posterior distribution of γ_a is Bernoulli with $p_{\gamma_a} = \frac{BF}{1+BF}$. Thus we can sample

$$\gamma_a \sim Ber(p_{\gamma_a}).$$

The Bayes factor BF is given by

$$BF = \frac{f(\mathbf{n}|\gamma_a = 1, \boldsymbol{\gamma}_{\setminus a}, \boldsymbol{\alpha}) pr_{\gamma_a}}{f(\mathbf{n}|\gamma_a = 0, \boldsymbol{\gamma}_{\setminus a}, \boldsymbol{\alpha})(1 - pr_{\gamma_a})}.$$

The parameters α_a, σ_a^2 and γ_a are updated in turn for all $a \subseteq C$. In this way we are able to efficiently sample from the full posterior $f(\boldsymbol{\alpha}, \boldsymbol{\gamma}, \boldsymbol{\sigma}^2|\mathbf{n})$ and derive from it the posterior of $f(\boldsymbol{\beta}, \boldsymbol{\gamma}, \boldsymbol{\sigma}^2|\mathbf{n})$. From the

marginal posterior distribution $f(\boldsymbol{\gamma}|\mathbf{n})$, we can estimate the model probabilities by the sample proportions for $\boldsymbol{\gamma}$, with the most promising models corresponding to the most frequently observed $\boldsymbol{\gamma}$. From $f(\boldsymbol{\beta}|\mathbf{n}, \gamma)$ we can derive the distribution for the interaction strength vector $\boldsymbol{\beta}$ conditional on the model $\boldsymbol{\gamma}$.

Hierarchical Bayesian model selection

We adapt the algorithm described above in a way that allows only moves from one hierarchical model to another, so that we never leave the class of hierarchical models. A hierarchical model is determined by its generators. The only individual model term which may be removed from a hierarchical model so that it remains hierarchical is a generating term. In addition, [7] define the dual generators, which are the minimal terms that are not present in the model. The only individual model terms which may be added to the model so that it remains hierarchical are the dual generators.

We consider all hierarchical models to be equally likely and denote the set of generators and dual generators of a hierarchical model corresponding to $\boldsymbol{\gamma}$ with $G_{\boldsymbol{\gamma}}$. We use a Metropolis Hastings algorithm to sample from the full posterior distribution $f(\boldsymbol{\gamma}, \boldsymbol{\alpha}, \boldsymbol{\sigma}^2|\mathbf{n})$. We propose a move from one model $\boldsymbol{\gamma}^t$ to the next model $\boldsymbol{\gamma}^{t+1}$ by choosing an element $G_{\boldsymbol{\gamma}^t}$. Thus we randomly sample an element $a \in G_{\boldsymbol{\gamma}^t}$ and the corresponding γ_a is set to one or zero respectively. The resulting $\boldsymbol{\gamma}$ is denoted as $\boldsymbol{\gamma}^{t+1}$. The corresponding move is accepted with acceptance probability:

$$\min\left(1, \frac{f(\mathbf{n}|\boldsymbol{\gamma}^{t+1}, \boldsymbol{\alpha}^t)|G_{\boldsymbol{\gamma}^t}|}{f(\mathbf{n}|\boldsymbol{\gamma}^t, \boldsymbol{\alpha}^t)|G_{\boldsymbol{\gamma}^{t+1}}|}\right),$$

where $|G|$ refers to the number of models included in each set of generators G and this refers to the probability of proposing each model. The sampling procedure for α_a and σ_a^2 is performed exactly as in the non-hierarchical case described in the previous section.

Prior specification for Bayesian methods

For the Bayesian estimation of the parameter vector, we must specify the parameters for the prior distribution of σ_a^2: σ_a^2 plays a role that is similar to that of the parameter λ in the Lasso. The lower σ_a^2, the smaller the estimated coefficient $\widehat{\beta}_a$. An empirical Bayes approach to the implementation could be to specify this parameter by cross-validation. While feasible for the ℓ_1-regularization approaches, cross-validation becomes prohibitive for the MCMC approaches because of the computational demands. [5] proposed a fixed value of two for all a in C, e.g. $\sigma_a^2 = \sigma^2$. Placing a normal prior with mean zero and variance two on each α_a means that with probability 0.95, each of these effects will increase or decrease the

ratio of any two cell probabilities by a factor of no more than 10. This is a relatively vague prior, and can be appropriate when no prior information is available. However, our simulation study will illustrate that the final results can be highly sensitive to the choice of this value. To mitigate this sensitivity, we assume σ^2 to have an inverse gamma distribution with mean and variance equal to one, as described in the *Model Selection* section.

In addition, for non-hierarchical model selection, we have to specify the prior distribution for γ_a. We set $\gamma_a \sim Ber(pr_{\gamma_a})$, where pr_{γ_a} reflects prior belief that the corresponding interaction term U_a is present. Without prior knowledge, we assume here that all possible models are a priori equally likely, corresponding to $pr_{\gamma_a} = 1/2$ for all $a \subseteq C$.

This prior is especially attractive when coupled with MAP estimation, as done here, because it effectively cancels out of the MAP calculation. In other situations, this prior may be less compelling. For example, it may be of interest to report posterior probabilities of properties of sets in the model space, such as marginal posteriors of the inclusion of certain coefficients or marginal posteriors of the presence of high order interactions. Then one has to evaluate carefully the mass that priors give to those sets, and one might have to reconsider the choice of the prior distributions to get reasonable posterior probabilities of these sets. In addition, as q, the number of exons, increases, estimating the MAP in the model space becomes difficult and marginal posteriors of summaries such as the model size or the maximum order of interaction may be all that can be reliably estimated. In those circumstances, we suggest graphing these posteriors along with the corresponding priors probabilities, and/or to report Bayes factors.

Lemma 1. *The function $f(\alpha_a|\mathbf{n}, \boldsymbol{\gamma}, \boldsymbol{\alpha}_{\setminus a})$ is log-concave for the prior distributions chosen as described in (1).*

Proof. Without loss of generality we assume that α_a is univariate. The proof for the case that α_a is a vector is exactly the same but for a single component of α_a. We have to prove that the function $h(\alpha_a)$ is concave for

$$h(\alpha_a) = n\alpha_\emptyset + \mathbf{n}^t X_a \alpha_a \gamma_a - \frac{1}{2\sigma^2}\alpha_a^2,$$

where $\alpha_\emptyset$ is the normalizing constant ensuring that all cell probabilities add up to one. This constant depends on α_a. As the last two terms are concave it remains to be shown that $n\alpha_\emptyset(\alpha_a)$ is concave. For $\gamma_a = 0$ this term is constant and $h(\alpha_a)$ is therefore concave. For $\gamma_a = 1$, we set $\boldsymbol{X}' = \boldsymbol{X}_{\setminus\emptyset}$ and $\boldsymbol{\alpha}' = \boldsymbol{\alpha}_{\setminus\emptyset}$, it

then holds

$$\begin{aligned} h(\alpha_a) &= n\alpha_\emptyset = -n\log\sum_{i=1}^{m}\exp\left((\boldsymbol{X}'\boldsymbol{\alpha}')_i\right), \\ h'(\alpha_a) &= -n\frac{X_a^t\exp\left(\boldsymbol{X}'\boldsymbol{\alpha}'\right)}{\sum_{i=1}^{m}\exp\left((\boldsymbol{X}'\boldsymbol{\alpha}')_i\right)}, \\ h''(\alpha_a) &= -n\frac{(X_a^2)^t\exp\left(\boldsymbol{X}'\boldsymbol{\alpha}'\right)\sum_{i=1}^{m}\exp\left((\boldsymbol{X}'\boldsymbol{\alpha}')_i\right)-(X_a^t\exp\left(\boldsymbol{X}'\boldsymbol{\alpha}'\right))^2}{(\sum_{i=1}^{m}\exp\left((\boldsymbol{X}'\boldsymbol{\alpha}')_i\right))^2}, \end{aligned}$$

where $\exp\left(\boldsymbol{X}'\boldsymbol{\alpha}'\right)$ has to be understood as the componentwise application of the exponential function and likewise for X_a^2. We now have to show that $h''(\alpha_a)$ is less than zero. If we denote $\exp\left(\boldsymbol{X}'\boldsymbol{\alpha}'\right)$ by $\mathbf{u}$ and X_a with $\mathbf{x}$, it is sufficient to prove that

$$\sum_{j=1}^{m}x_j^2u_j\sum_{i=1}^{m}u_i-(\mathbf{x}^t\mathbf{u})^2\geq 0.$$

The above expression is

$$\sum_{\substack{i,j\\ j<j}}((x_j^2u_ju_i+x_i^2u_iu_j)-(2x_iu_ix_ju_j))=\sum_{\substack{i,j\\ j<j}}(x_j^2+x_i^2-2x_ix_j)u_iu_j=\sum_{i,j,i<j}(x_j-x_i)^2u_iu_j,$$

which is greater than zero, as $\mathbf{u}>0$. This proves Lemma 1. □

Additional file 3 – Additional datasets

We consider two cDNA libraries from two different developmental stages of human brain, for the gene *CACNA1G* encoding the low voltage-activated calcium channel gene $\mathrm{Ca}_v3.1$. This gene is known to be alternatively spliced at $q=9$ sites. Detailed information on this dataset can be found in Emerick *et al.* (2006).

Results

We estimate the interaction pattern $\widehat{\boldsymbol{\beta}}$ with the step ℓ_1-regularization method. For the fetal as well as for the adult tissue, a model involving first order interactions only is estimated. By looking at the interaction graphs in Figure 1, we clearly see that the patterns exhibit differences. While in the fetal tissue, exon eight interacts with most other exons, this is no more the case for adult tissue where exon number five seems to play a key role. Exon five and eight correspond to exon 30B and exon 35 in Emerick *et al.* (2006).

We already know that exon 30B plays a key role, because the deletion of segment 30B causes a frameshift, resulting in premature chain termination caused by an early stop codon downstream of this splice site in

the new translation reading frame. Transcripts with this condition are often eliminated before they can be translated into proteins through a process called nonsense-mediated decay (NMD). Thus the frequency of these splice variants in the cDNA library may significantly under-represent their rate of production through transcription and splicing. NMD is an efficient way to use alternative splicing to turn off expression of a single gene in a specific class of cells, without altering gene expression in ways that might effect other genes or even the same gene in neighboring cells of the same tissue. This type of activity shows as a fairly high degree of splicing interactions between this site and other sites, reflecting splicing details in the particular classes of cells where this gene is inactivated by NMD.

References

1. Kuo L, Mallick B: **Variable Selection for Regression models**. *Sankhya B* 1998, **60**:65–81.
2. Ntzoufras I, Forster J, Dellaportas P: **Stochastic search variable selection for log-linear models**. *Journal of Statistical Computation and Simulation* 2000, :23–37.
3. George EI, McCulloch RE: **Variable selection via gibbs sampling**. *Journal of the American Statistical Association* 1993, **88**:881–889.
4. Geweke JF: **Variable selection and model comparison in regression**. Working Papers 539, Federal Reserve Bank of Minneapolis 1994. [Available at http://ideas.repec.org/p/fip/fedmwp/539.html].
5. Dellaportas P, Forster J: **Markov chain Monte Carlo model determination for hierarchical and graphical log-linear models**. *Biometrika* 1999, **86**:615–633.
6. Gilks WR, Wild P: **Adaptive rejection sampling for Gibbs sampling**. *Applied Statistics* 1992, **41**:545–557.
7. Edwards D, Havranek T: **A fast procedure for model search in multidimensional contingency tables**. *Biometrika* 1985, **72**:339–351.

Figure 1: Top: Comparison between the interaction pattern $\widehat{\boldsymbol{\beta}}$ of fetal and adult tissue. Below: Independence graphs of the estimated log-linear models. On the left are the estimated models, on the right with the strongest interactions only. Within an interaction degree, the sequence of coefficients is ordered from left to right as follows: e.g. for 2nd order interactions, $123, 124, 125, \ldots, 789$, where $1, \ldots, 9$ represent exons 25A, 14, 25B, 26, 30B, 31A, 34, 35, 38B as described in Emerick *et al.* (2006). We clearly see differences between fetal and adult tissue, especially if we look at the reduced models.

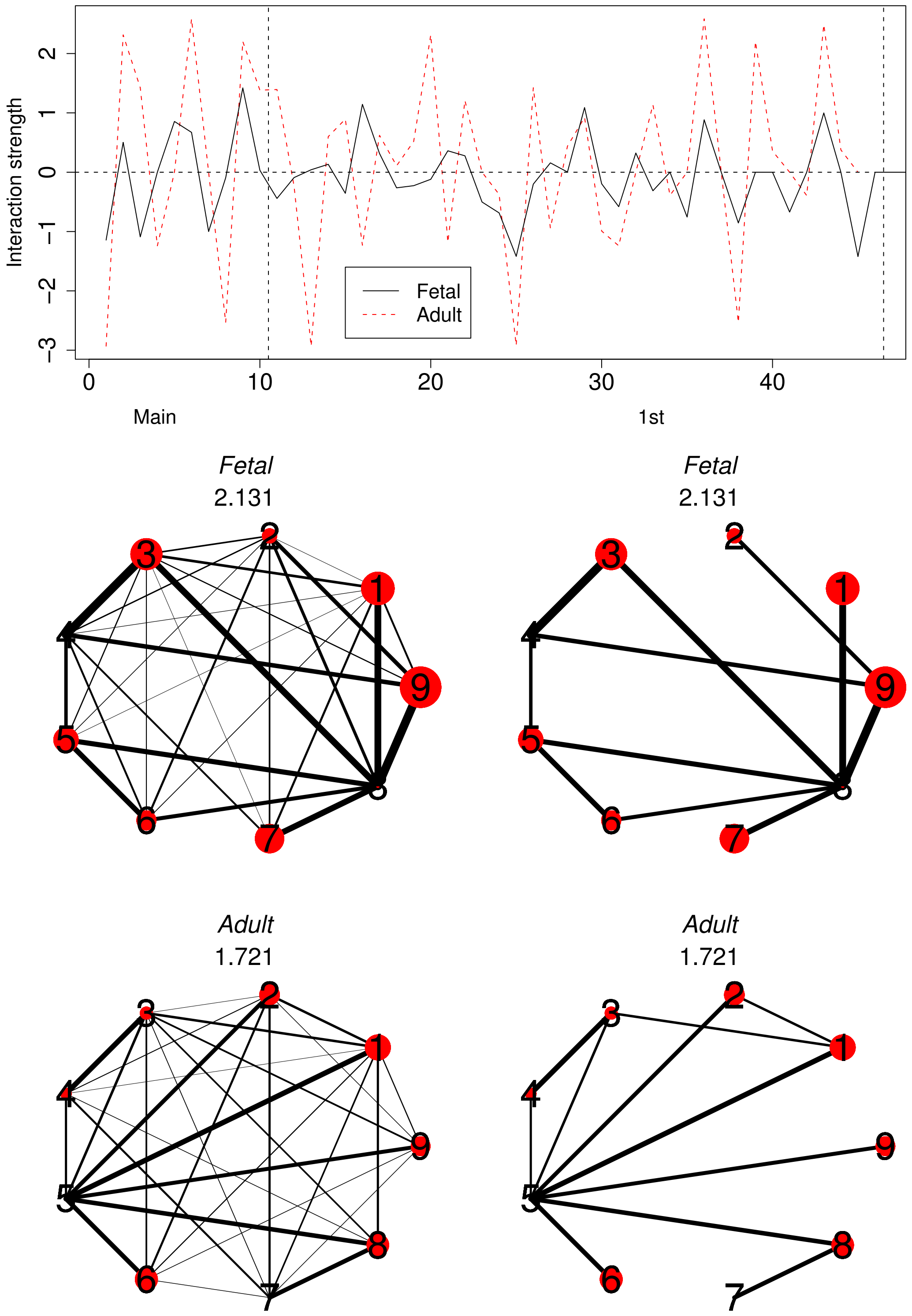

Supplement: Additional file 1 — The Additional file consists of 3 sections. Section 1 contains details concerning the parametrization of the log-linear model. Section 2 describes some Bayesian model selection approaches, which were used for comparison with our algorithm. In Section 3 a further dataset on which we tested our algorithm is introduced and the results are given on that dataset. [file 1471-2105-8-476-S1.pdf]
